# A Holistic Approach to Chronic Disease Prevention: Good Health and Wellness in Indian Country

**DOI:** 10.5888/pcd16.190081

**Published:** 2019-08-01

**Authors:** Nancy S. Andrade, David K. Espey, Mary E. Hall, Ursula E. Bauer

**Affiliations:** 1 National Center for Chronic Disease Prevention and Health Promotion, Centers for Disease Control and Prevention, Atlanta, Georgia

## Abstract

The National Center for Chronic Disease Prevention and Health Promotion at the Centers for Disease Control and Prevention funds the agency’s largest investment in Indian Country, Good Health and Wellness in Indian Country. This 5-year program, launched in 2014, supports American Indian and Alaska Native communities and tribal organizations to address chronic diseases and risk factors simultaneously and in coordination. This article describes the development, funding, and implementation of the program. Dialogue with tribal members and leaders helped shape the program, and unlike previous programs that funded a small number of tribes to work on specific diseases, this program funds multiple tribal entities to reach widely into Indian Country. Implementation included culturally developed and adapted practices and opportunities for peer sharing and problem solving. This program identified approaches useful for the Centers for Disease Control and Prevention, other federal agencies, or other organizations working with American Indians and Alaska Natives.

**Summary**

**What is already known on this topic?**

Federal agencies typically fund and support only a small number of federally recognized tribes to address chronic diseases and require grantees to focus on evidence-based approaches, without flexibility for cultural adaptation and approaches aligned with tribal practices.

**What is added by this report?**

We implemented an innovative, holistic approach to supporting scores of tribes to apply effective strategies by building on tribal practices that keep people well and adapting Western chronic disease prevention programs.

**What are the implications for public health practice?**

This program offers a model that other agencies could adapt to support tribes and tribal organizations to build on their cultural strengths and more effectively prevent disease and promote health.

## Background

American Indian and Alaska Native communities have strengths retained and adapted from their culture over thousands of years. They also share a history of adversity from centuries of European colonization (1). This adversity is among the reasons why good health and wellness have eluded many American Indians and Alaska Natives. Despite 20th-century improvements in water, sanitation, control of infectious diseases, and improved nutrition in American Indian and Alaska Native communities, new challenges to health have emerged, reflecting both the legacy of colonization and prevailing trends in the wider society, including changes in diet and physical activity patterns and use of commercial tobacco and alcohol. Obesity is common in many American Indian and Alaska Native communities (2). Obesity has contributed to the epidemic of type 2 diabetes (3), with widespread consequences for individuals and families living in these communities. Commercial tobacco use is prevalent, particularly among Great Plains tribes and Alaska Native villages (4), leading to high rates of cancer (5), heart disease (6) and stroke (7). American Indians and Alaska Natives have the highest rate of chronic liver disease linked to alcohol misuse and viral hepatitis (8), as well as high rates of substance abuse (9), unemployment (10), despondency (11), domestic violence (12), mental illness (13) and suicide (14). To build healthier native communities, in light of these many health challenges, tribal elders and health leaders have sought to draw on strong connections to culture, heritage, and community and pair them with funding and support for culturally appropriate programs from federal agencies. (15). The Substance Abuse and Mental Health Services Administration’s National Tribal Behavioral Health Agenda is a case in point, created with extensive input from tribal leaders and in collaboration with governmental and nongovernmental organizations. This approach uses tribal cultural practices, efforts to connect American Indians and Alaska Natives to culture and heritage, and Western evidence-based practices to improve health and wellness in partnership with native communities (11).

Similar to other federal agencies, the Centers for Disease Control and Prevention (CDC) provides funding for health promotion and disease prevention programs through competitive grants and cooperative agreements to states and to smaller jurisdictions, such as cities and counties, and to federally recognized tribes. However, federal agencies are challenged to provide funding and support to a large proportion of tribes because of the sheer number of federally recognized tribes — currently 573 — eligible for such assistance. The result is that many funding opportunities for which tribes are eligible usually are awarded to only a small proportion of tribes. In addition, these funding opportunities typically have not permitted adaptation of supported approaches to the unique and diverse cultures and traditions in Indian Country. This lack of permitted adaptation has limited tribal interest in applying for funding and in the utility and effectiveness of the funding when awarded. In this article, we describe CDC’s Good Health and Wellness in Indian Country (GHWIC) program, which offers a holistic, coordinated approach to chronic disease prevention and health promotion, including culturally adapted approaches for American Indian and Alaska Native communities (16,17), and supports a larger number of tribes (more than 100) than has typically been supported (fewer than 10).

## History of NCCDPHP’s Tribal Portfolio and Evolution of GHWIC

CDC’s National Center for Chronic Disease Prevention and Health Promotion (NCCDPHP) has invested in public health programs in Indian Country since the early years of the center’s creation in 1988. Until 2014, these programs funded small numbers of tribes and tribal organizations for disease-related prevention and control activities, and focused first on cancer prevention and control, then type 2 diabetes, and more recently on tobacco use prevention and control. These investments in tribes and tribal organizations accomplished important work. However, these efforts were smaller and less coordinated than is desirable given the large number, diversity, and geographic spread of tribes and tribal organizations, and the breadth of chronic disease prevention and control work to be done. Thus, these efforts struggled to achieve measurable, population-wide progress on chronic disease outcomes.

For 5 years, from 2008 to 2013, with financial support from the Indian Health Service (IHS), NCCDPHP shifted the paradigm with the launch of the Traditional Foods program, designed to resurrect and improve healthy traditional diets to prevent and manage type 2 diabetes (18). The program was groundbreaking in 3 ways: it drew on indigenous knowledge and supported indigenous ways of living to protect and promote health; it funded a larger number of tribes than had been previously funded (17); and it worked more aggressively to disseminate successful practices beyond grantees in a single 5-year program (18). The program expanded the cultivation of healthy foods and revived and extended skills in traditional food gathering, production, and storage. Ultimately, the Traditional Foods program led the way for Good Health and Wellness in Indian Country (GHWIC) (19), a new NCCDPHP program, grounded in a collaborative approach to working with tribes, villages, and tribal organizations, and drawing on traditional tribal practices.

A 5-year program that began in late 2014, GHWIC implemented lessons from the Traditional Foods program, including supporting culturally developed and adapted practices and working to disseminate approaches and practices in an IHS service area and across Indian country. GHWIC was also shaped by several years of listening to and dialogue with CDC/Agency for Toxic Substances and Disease Registry’s (ATSDR’s) Tribal Advisory Committee, and listening sessions and visits with tribes and tribal leaders in Indian Country. The program sought to address key lessons from these encounters: incorporation of tribal wisdom to protect and promote physical, mental, and spiritual wellbeing; avoidance of a disease-management approach to instead work upstream addressing drivers of poor health through culturally appropriate practices; and solving the problem of funding only a small number of tribes to do disease-specific work in favor of a holistic approach that reaches deeply and widely into Indian Country.

## GHWIC Program Design, Funding, and Implementation

Four divisions in NCCDPHP contributed funds to the GHWIC Program. The Division for Heart Disease and Stroke Prevention and the Division of Diabetes Translation each provided $6 million, the Office on Smoking and Health provided $2.8 million, and the Division of Nutrition, Physical Activity, and Obesity provided $1 million. Because chronic diseases such as heart disease and type 2 diabetes are driven, in large part, by poor nutrition, lack of physical activity and tobacco use, awardees were able to use program funds from the 4 divisions to implement effective, culturally appropriate interventions to improve these health behaviors and contribute to long-term disease prevention outcomes (16,17). Awardees developed and implemented a single, cohesive work plan — instead of 4 separate work plans — that invested a critical mass of resources in a holistic set of interventions to prevent chronic disease and promote health, including reviving traditional healthy foods, physical activity, and connecting tribal members to community and culture to support healthy behaviors.

The GHWIC program funded tribes, villages, tribal organizations, and Tribal Epidemiology Centers (TECs) through a tiered approach to maximize funds throughout Indian Country. Twelve tribes were funded to use community-chosen and culturally adapted policies, systems, and environmental improvements to achieve GHWIC’s long-term goals to reduce the prevalence of obesity, death, and disability from tobacco use, type 2 diabetes, heart disease, and stroke. Eleven tribal organizations provided leadership, technical assistance, and resources to more than 100 tribes and tribal organizations in their IHS administrative areas and to funded tribes (20). Eleven TECs served tribes, villages, and tribal organizations in each IHS administrative area to evaluate GHWIC interventions and demonstrate program impact. One TEC, the Urban Indian Health Institute, coordinated the national evaluation by providing evaluation expertise to other TECs, tribal organizations, tribes, and CDC (20).

Providing opportunities for problem solving and sharing knowledge among peers was a priority for the program. Awardees planned 2 resource meetings, which were critical to developing the grantee-driven identity of GHWIC and to position grantees as resources, mentors, and guides for each other. CDC and awardees jointly planned a midpoint meeting in which awardees presented and reflected on their work, discussed program midpoint outcomes, and received training and technical assistance from CDC and each other. CDC adapted the Extension for Community Healthcare Outcomes (ECHO) model to build and support a community of practice among GHWIC awardees (21). The ECHO model is an evidence-based education, workforce development, and collaborative problem-solving intervention that strengthens knowledge and practice in the field (22). Although the ECHO model was originally developed to improve health care practice, CDC recognized an opportunity to adapt the model to a new public health context in Indian Country, as a way to foster knowledge sharing and peer-to-peer technical assistance among grantees. GHWIC ECHO sessions are culturally tailored by using awardee guidance to create opportunities for peer-to-peer learning and collaboration. The model develops knowledge and capacity among awardees, using technology (multipoint video conferencing and internet) to facilitate sharing best practices and troubleshooting challenges, providing case-based learning through awardee presentations and providing technical assistance and training from subject matter experts.

As a result of ECHO learning and sharing, awardees are more effectively implementing policy, systems, and environmental changes to prevent chronic disease in their communities (21). CDC’s GHWIC program was one of the first adaptations of the ECHO model to a public health context that identified and disseminated ideas, innovation, wisdom, and practice across a group of peers, with less direct knowledge dissemination from the “hub” (CDC) to the “spokes” (the awardees). Instead, awardees regularly exchanged roles, serving as spokes or hubs, depending on topic and experience.

## Implication for Public Health Practice

GHWIC is CDC’s largest investment to date in Indian Country and the first CDC funding opportunity designed to support American Indian and Alaska Native communities and tribal organizations to address multiple chronic diseases and risk factors simultaneously, and in coordination. Developed and implemented following the recommendations and requests of the CDC/ATSDR’s Tribal Advisory Committee, GHWIC is an example of CDC’s commitment to support chronic disease prevention and health promotion in collaboration with tribal partners. GHWIC provides a rich opportunity to identify approaches that might be useful for CDC, other federal agencies, or other organizations to be more effective working in or planning to work in Indian Country.

Key among the lessons learned through the implementation of GHWIC is the approach of funding administration-area–level tribal organizations that can expand the reach of limited resources to many more tribal partners than is possible by funding individual tribes directly. More than 100 tribes received CDC funding indirectly through GHWIC recipients (20). This model is expected to have benefits beyond simply serving as a mechanism to increase the reach of limited funding. By supporting an administration area or regional tribal organization to provide leadership, resources, technical assistance, and evaluation support, the tribal capacity and infrastructure gained are local and more tailored to the needs of local tribes. Increased funding for administration-area–level partners also provides employment and workforce development opportunities for local tribal members, as well as practical experience in chronic disease prevention and wellness promotion.

CDC and other federal agencies are accustomed to using uniform approaches to support states, tribes, local jurisdictions, and territories in addressing diseases or risk factors in categorical funding announcements (23). These approaches can be barriers to more integrated, customized, and culturally relevant efforts to prevent chronic disease and promote wellness. By combining funding from several divisions at CDC, GHWIC was able to encourage tribal partners and CDC staff members who supported them to take a more integrated approach in the development and implementation of chronic disease prevention programs that share common risk factors (eg, obesity, tobacco use, lack of physical activity). However, CDC program staff members note that practical challenges remain. Providing coordinated and consistent technical support across multiple program areas continues to be a challenge, even as doing so is critical to support a more effective approach and is a more effective model of collaboration with tribal partners.

As CDC staff members observed, some tribal partners, particularly those unaccustomed to CDC cooperative agreements, struggled to meet requirements of federal grants. Despite great health needs and concerns in their communities, some grantees were unable to spend their award in a timely fashion for reasons including time-consuming approval processes in their tribes, difficulty recruiting staff, staff turnover at CDC or among tribal partners, and difficulty deciphering complex federal reporting requirements. These challenges are not unique to tribal awardees, but they must be attended to and might require additional assistance, flexibility, and new approaches to support tribal partners and achieve success.

Finally, GHWIC established a foundation for CDC to build on in 2 new initiatives to support tribes and tribal partners in chronic disease prevention, health promotion, and other health programs in Indian Country. One program, launched in 2017, funds the TECs to strengthen their public health capacity and to better support the public health needs of the tribes in their regions (24) and is funded at $42 million for 5 years. The second program is Tribal Practices for Wellness in Indian Country (TPWIC), a $15 million 3-year program, launched in 2018, that provides funding to 21 tribes and 16 urban Indian organizations. This program incorporates approaches and strategies identified by American Indian and Alaska Native leaders and supports cultural and traditional practices that build strength and resilience and support healthy behaviors (15).

Implementation of GHWIC marked an important shift in CDC’s support of American Indian tribes and Alaska Native villages as they strive to prevent chronic disease and promote health in ways that are relevant to them. By deploying a holistic approach to address multiple chronic diseases and risk factors simultaneously, by expanding the reach of limited funding through tiered funding and subawards, by supporting tribal practices that keep people well, and by adapting Western chronic disease prevention approaches, GHWIC offers a new model for health and wellness in Indian Country. Programs such as GHWIC support tribes and tribal organizations to build on their cultural strengths and more effectively address threats to health and wellness in Indian Country.
